# Vitamin C in Sepsis: Exploring Its Role in Modulating Inflammation and Oxidative Stress

**DOI:** 10.7759/cureus.81386

**Published:** 2025-03-28

**Authors:** Kashmala Wazir, Muhammad Abubakar, Ayisha Anwar, Farah Rahat Rashid, Afshan Adil, Muhammad Sajjad

**Affiliations:** 1 Medicine, Lady Reading Hospital Peshawar, Peshawar, PAK; 2 Internal Medicine Residency Program, Medical Education, Hamad General Hospital, Doha, QAT; 3 Medicine and Surgery, Al Abeer Medical Centre, Doha, QAT; 4 Medicine, Khyber Teaching Hospital, Peshawar, PAK; 5 Medicine, Hamad General Hospital, Doha, QAT; 6 Biological Sciences, Kohat University of Science and Technology, Kohat, PAK

**Keywords:** clinical outcomes, inflammation, oxidative stress, sepsis, vitamin c

## Abstract

Background

Despite improvements in medical treatment, sepsis remains a deadly illness with high fatality rates that is characterized by excessive inflammation and oxidative stress.

Objective

This study aimed to evaluate the impact of vitamin C supplementation on inflammatory response, oxidative stress, and clinical outcomes in sepsis patients.

Methodology

A prospective observational study was conducted over one year at Lady Reading Hospital, Peshawar, including 326 adult sepsis cases meeting the Sepsis-3 criteria. To maximize early therapeutic benefits, intravenous vitamin C supplementation (1,500 mg/day) was administered for 14 days, followed by a maintenance regimen of 1,500 mg/day for 14 days every 3-6 months to sustain therapeutic effects. The efficacy of vitamin C was assessed through serial measurements of oxidative stress markers (malondialdehyde) and inflammatory markers (CRP, IL-6, and TNF-α) at baseline and at 3, 6, 9, and 12 months. Clinical outcomes, including ICU stay, organ dysfunction, and mortality, were prospectively recorded. Statistical analysis was performed using SPSS version 25 (IBM Corp., Armonk, NY, USA).

Results

Vitamin C supplementation in sepsis patients led to significant improvements over 12 months, with reductions in IL-6 (345.16 ± 128.52 to 245.12 ± 83.78 pg/mL), C-reactive protein (CRP) (175.31 ± 62.43 to 114.57 ± 38.67 mg/L), and malondialdehyde (MDA) levels (8.94 ± 3.21 to 5.79 ± 2.18 nmol/mL). Clinical outcomes also improved, including a decrease in the 30-day mortality rate from 14.11% (n=46) to 6.90% (n=22), organ dysfunction score from 9.82 ± 4.03 to 4.83 ± 2.54, and ICU stay from 11.57 ± 3.85 to 7.24 ± 3.01 days. Regression analysis indicated that vitamin C supplementation was significantly associated with better outcomes (β = -2.34, p = 0.012), while chronic kidney disease was associated with worse outcomes (β = 1.75, p < 0.001).

Conclusion

Supplementing with vitamin C has been shown to significantly improve clinical outcomes in sepsis by lowering oxidative stress and inflammation.

## Introduction

Sepsis is a potentially fatal illness that develops when the body's reaction to an infection results in extensive inflammation, which damages tissue, causes organ failure, and often results in death [1]. Despite improvements in medical treatment, it continues to pose a serious threat to world health due to its high fatality rates [2]. Improving patient outcomes requires early detection and efficient treatment [3]. The quest for adjuvant medicines that alter the host's immune response and enhance clinical outcomes is still continuing, even though current therapeutic methods concentrate on managing the underlying infection and promoting organ function [4].

Ascorbic acid, another name for vitamin C, is a vital nutrient with strong antioxidant qualities. It is essential for several physiological functions, such as collagen formation, immunological response, and cellular defense against oxidative stress [5,6]. Recent research indicates that since vitamin C may enhance vascular function, lower oxidative damage, and control the inflammatory response, it may have therapeutic promise in sepsis [7]. These consequences are especially pertinent in sepsis, since endothelial dysfunction, oxidative stress, and excessive inflammation are major factors in the development of the illness and organ failure [8].

Vitamin C administration may enhance clinical outcomes in sepsis, including decreasing death rates, reducing organ dysfunction, and shortening hospital stays, according to recent research [9]. The results are still mixed, however, with some research revealing little to no advantages. These disparities might be caused by variations in patient groups, dosage schedules, and research designs [10]. Critical areas of continuing study include figuring out the best dosage regimens and comprehending the underlying processes via which vitamin C acts in sepsis [11].

The purpose of this study is to look at how vitamin C affects oxidative stress, inflammatory markers, and clinical outcomes in sepsis. This research aims to add to the expanding body of knowledge that might guide clinical practice and enhance patient care by shedding light on the possible therapeutic advantages and mechanisms of vitamin C in sepsis.

## Materials and methods

Study design and setting

This study was a prospective observational study conducted at Lady Reading Hospital Peshawar over a duration of one year, from January 2020 to December 2020.

Inclusion and exclusion criteria

Adult patients aged 18 years or older who were admitted to the intensive care unit (ICU) with a diagnosis of sepsis based on the Sepsis-3 criteria and required routine sepsis therapy were included in the study. The age cutoff was chosen because the Sepsis-3 criteria are primarily validated in adult populations, and pediatric sepsis follows different pathophysiological mechanisms and treatment protocols. Patients were excluded if they had a known allergy to vitamin C supplements, chronic renal failure requiring dialysis, were pregnant or nursing, or refused to provide informed consent.

Sample size

A total of 326 patients meeting the inclusion criteria were included in the study. Consecutive sampling was used, meaning that all eligible sepsis patients admitted to the ICU during the study period were enrolled without pre-selection. This approach minimized selection bias and ensured a representative sample, enhancing the generalizability of the findings.

Data collection

Patient data were collected using a structured proforma. Upon admission, baseline laboratory tests, clinical characteristics, and demographic information were recorded. Patients were monitored for oxidative stress markers, including malondialdehyde, and inflammatory markers such as C-reactive protein (CRP), interleukin-6 (IL-6), and tumor necrosis factor-alpha (TNF-α). Clinical outcomes, including ICU stay, organ dysfunction scores, and mortality rates, were assessed prospectively. ICU stay was documented at admission and reassessed during follow-up at 3, 6, 9, and 12 months to track patients requiring re-admission due to sepsis-related complications, ensuring a comprehensive evaluation of ICU utilization over time.

To maximize early therapeutic benefits, vitamin C supplementation was administered intravenously at 1,500 mg/day for 14 days. This dosing was based on pharmacokinetic studies demonstrating that intravenous vitamin C at this concentration achieves plasma levels sufficient to exert antioxidant and anti-inflammatory effects in critically ill patients [12-14]. The long-term maintenance regimen was revised to address reviewer concerns regarding evidence gaps. Instead of the previously described 1,500 mg every 3-6 months, a subgroup of patients who exhibited sustained oxidative stress and inflammatory marker elevation at follow-up received an additional 1,500 mg/day for another 7-14 days, based on clinical discretion and biomarker trends.

Standardization of concurrent sepsis therapies

To minimize potential confounding, all patients received standardized sepsis management based on the Surviving Sepsis Campaign (SSC) guidelines [15]. Broad-spectrum antibiotics were administered within one hour of sepsis diagnosis and adjusted based on culture sensitivity results. Vasopressors, primarily norepinephrine, were initiated if mean arterial pressure (MAP) fell below 65 mmHg. Fluid resuscitation was provided using balanced crystalloids, specifically Ringer’s lactate at 30 mL/kg for initial resuscitation, followed by goal-directed therapy based on lactate clearance and hemodynamic response. For patients requiring mechanical ventilation, lung-protective strategies were applied in cases of acute respiratory distress syndrome (ARDS). These standardized treatment protocols ensured that observed effects on oxidative stress, inflammation, and clinical outcomes were not attributable to variations in sepsis management strategies.

Statistical analysis and handling of missing data

Statistical analyses were performed using SPSS software version 25.0 (IBM Corp., Armonk, NY, USA). Continuous variables were expressed as mean ± standard deviation (SD) and compared using independent t-tests for normally distributed data, while categorical variables were presented as frequencies and percentages and analyzed using the chi-square (χ²) test. t-values and χ² values were reported to provide statistical comparisons across different follow-up periods. To identify independent determinants of clinical outcomes, multivariate regression analysis was conducted, adjusting for confounders such as age, comorbidities, baseline Sequential Organ Failure Assessment (SOFA) scores, and concurrent therapies.

To address missing data, particularly due to mortality-related attrition, a last observation carried forward method was applied for non-survivors up to their last recorded follow-up. Sensitivity analyses were conducted to determine whether excluding missing data significantly altered the study’s findings. This ensured that attrition did not introduce bias into the study’s conclusions regarding the impact of vitamin C on sepsis outcomes.

Ethical approval

Ethical clearance for the study was granted by the MTI-LRH Institutional Review Board (IRB). Written informed consent was obtained from all participants or their legal representatives before enrollment. To maintain patient confidentiality, only patient IDs were recorded, with no names or personal identifiers included in the dataset. All data were de-identified before analysis, and no patient identities were disclosed in the submitted manuscript. The data were securely stored in a password-protected electronic database, with restricted access granted only to authorized research personnel. The study adhered strictly to institutional ethical guidelines and data privacy regulations.

## Results

The baseline clinical and demographic features of sepsis patients are shown in Table 1. The patients' average age was 51.21 ± 14.35 years, and 129 (39.58%) of them were between the ages of 31 and 50. Of the patients, 132 (40.49%) were female and 194 (59.51%) were male. A significant degree of severity was indicated by the mean APACHE II score of 20.47 ± 4.72 and the mean Sequential Organ Failure Assessment (SOFA) score of 9.21 ± 3.17. Hypertension (n: 120; 36.80%), diabetes (n: 98; 30.06%), chronic renal disease (n: 72; 22.08%), and chronic liver disease (n: 54; 16.56%) were among the common comorbidities. These baseline attributes demonstrate the intricacy and gravity of the patient group under investigation.

**Table 1:** Baseline Demographic and Clinical Characteristics of Patients with Sepsis SOFA Score: Sequential Organ Failure Assessment Score; APACHE II Score: Acute Physiology and Chronic Health Evaluation II Score; ​​​​​​​BMI: Body Mass Index; ​​​​​​​IQR: Interquartile Range; ​​​​​​​SD: Standard Deviation

Variable	Category	Number of Patients (n; %)	Median (IQR)	Mean ± SD	p-value
Age (years)	18 - 30	97 (29.75%)			0.067
	31 - 50	129 (39.58%)			
	51 - 70	86 (26.38%)			
	> 70	14 (4.29%)			
	Total	326 (100%)	50 (38–63)	51.21 ± 14.35	
Gender	Male	194 (59.51%)	-	-	0.142
	Female	132 (40.49%)	-	-	
BMI (kg/m²)	-	-	27.8 (24.2–30.5)	27.1 ± 3.9	-
SOFA Score	-	-	9 (7–11)	9.21 ± 3.17	-
APACHE II Score	-	-	20 (17–23)	20.47 ± 4.72	-
Comorbidities	Hypertension	120 (36.80%)	-	-	-
	Diabetes	98 (30.06%)	-	-	-
	Chronic Kidney Disease	72 (22.08%)	-	-	-
	Chronic Liver Disease	54 (16.56%)	-	-	-

The oxidative stress, inflammatory, and clinical consequences of sepsis patients are shown in Table 2. The C-reactive protein (CRP) level was 175.31 ± 62.43 mg/L on average, which indicates a significant amount of inflammation. Significant inflammatory activity was suggested by the mean levels of pro-inflammatory cytokines TNF-α and IL-6, which were 345.16 ± 128.52 pg/mL and 85.29 ± 32.67 pg/mL, respectively. Oxidative stress was indicated by the mean malondialdehyde (MDA) level, which was 8.94 ± 3.21 nmol/mL. The average length of stay in the intensive care unit was 11.57 ± 3.85 days, and the average organ dysfunction score was 9.82 ± 4.03. Given the severity of sepsis and its effect on clinical outcomes, the 30-day death rate was 14.11% (n=46).

**Table 2:** Inflammatory, Oxidative Stress Markers, and Clinical Outcomes in Sepsis Patients C-Reactive Protein (CRP): A marker of systemic inflammation, with higher levels indicating increased inflammatory activity. Interleukin-6 (IL-6): A pro-inflammatory cytokine involved in immune response regulation; elevated levels reflect severe inflammation. Tumor Necrosis Factor-Alpha (TNF-α): A key inflammatory mediator contributing to immune system activation and sepsis progression. Malondialdehyde (MDA): A biomarker of oxidative stress, indicating lipid peroxidation and cellular damage. Organ Dysfunction Score: A composite measure used to assess the severity of organ failure in sepsis. Intensive Care Unit (ICU) Stay: The duration (in days) that patients remain in the ICU for sepsis management. Mortality Rate: The percentage of patients who did not survive within 30 days of sepsis onset. Mean ± Standard Deviation (SD): Represents the average value of a variable along with its variability. 95% Confidence Interval (CI): The statistical range within which the true population value is expected to fall with 95% certainty. Picograms per milliliter (pg/mL): A unit of measurement for cytokine levels in the blood. Nanomoles per milliliter (nmol/mL): A unit of measurement indicating oxidative stress markers in the blood.

Variable	Category	Mean ± SD / Frequency (n; %)	Reference Range	95% CI
CRP (mg/L)	Mean ± SD	175.31 ± 62.43	< 10 mg/L	160.45 – 190.17
Pro-Inflammatory Cytokines	IL-6 (pg/mL)	345.16 ± 128.52	< 7 pg/mL	318.21 – 372.11
	TNF-α (pg/mL)	85.29 ± 32.67	< 8 pg/mL	78.52 – 92.06
Oxidative Stress Marker	MDA (nmol/mL)	8.94 ± 3.21	1.0 – 4.0 nmol/mL	8.12 – 9.76
Organ Dysfunction Score	Mean ± SD	9.82 ± 4.03	-	8.97 – 10.67
ICU Stay (days)	Mean ± SD	11.57 ± 3.85	-	10.78 – 12.36
Mortality Rate (n; %)	30-day mortality	46 (14.11%)	-	10.45 – 17.77%

The clinical outcomes and follow-up evaluations of sepsis patients over a one-year period are shown in Table 3. At baseline, the ICU stay was 11.57 ± 3.85 days, the organ dysfunction score was 9.82 ± 4.03, and the 30-day mortality rate was 14.11% (n=46). At three months, significant improvements were observed, with a decrease in ICU stay to 9.31 ± 3.17 days (p = 0.015) and a reduction in organ dysfunction score to 7.86 ± 3.52 (p = 0.009). The mortality rate also declined to 12.50% (n=41, p = 0.032).

**Table 3:** Follow-up Assessments and Clinical Outcomes t-values correspond to paired t-tests for continuous variables. χ² (chi-square) values are reported for categorical variables. *p-values < 0.05 indicate statistical significance. Effect size (Cohen’s d): Small (0.2–0.4), Medium (0.5–0.7), Large (>0.8). 95% CI for mortality rates provides more precise estimations.

Follow-Up Period	Clinical Outcomes	Mean ± SD / Frequency (n; %)	Test Statistic	Effect Size (Cohen's d)	95% CI	p-value
Baseline	ICU Stay (days)	11.57 ± 3.85	-	-	-	-
	Organ Dysfunction Score	9.82 ± 4.03	-	-	-	-
	Mortality Rate (n; %)	46 (14.11%)	-	-	(10.3%–18.6%)	-
3 Months	ICU Stay (days)	9.31 ± 3.17	t = 2.45	0.42	-	0.015*
	Organ Dysfunction Score	7.86 ± 3.52	t = 2.67	0.48	-	0.009*
	Mortality Rate (n; %)	41 (12.50%)	χ² = 4.62	-	(9.2%–16.5%)	0.032*
6 Months	ICU Stay (days)	8.72 ± 3.23	t = 2.34	0.51	-	0.021*
	Organ Dysfunction Score	6.93 ± 3.15	t = 2.59	0.57	-	0.011*
	Mortality Rate (n; %)	34 (10.30%)	χ² = 4.89	-	(7.4%–14.2%)	0.027*
9 Months	ICU Stay (days)	7.95 ± 3.32	t = 2.56	0.59	-	0.012*
	Organ Dysfunction Score	5.68 ± 2.87	t = 2.72	0.65	-	0.008*
	Mortality Rate (n; %)	28 (8.70%)	χ² = 5.31	-	(5.9%–12.4%)	0.019*
12 Months	ICU Stay (days)	7.24 ± 3.01	t = 2.89	0.72	-	0.007*
	Organ Dysfunction Score	4.83 ± 2.54	t = 2.98	0.78	-	0.005*
	Mortality Rate (n; %)	22 (6.90%)	χ² = 5.78	-	(4.5%–10.3%)	0.014*

By six months, further reductions were noted, with ICU stay decreasing to 8.72 ± 3.23 days (p = 0.021), organ dysfunction score improving to 6.93 ± 3.15 (p = 0.011), and mortality dropping to 10.30% (n=34, p = 0.027). At nine months, the ICU stay reduced to 7.95 ± 3.32 days (p = 0.012), organ dysfunction score improved to 5.68 ± 2.87 (p = 0.008), and the mortality rate declined to 8.70% (n=28, p = 0.019). By 12 months, the ICU stay further decreased to 7.24 ± 3.01 days (p = 0.007), the organ dysfunction score improved significantly to 4.83 ± 2.54 (p = 0.005), and the mortality rate reached 6.90% (n=22, p = 0.014). These findings demonstrate a consistent and statistically significant improvement in clinical outcomes over time, supporting the role of vitamin C supplementation in sepsis management (Table 3).

The chi-square test findings for the study's categorical variables are shown in Table 4. Sepsis results were not substantially correlated with gender (p = 0.170). Mortality (30-day) revealed a very significant connection (p < 0.001*), however, comorbidities such as Chronic Kidney Disease showed a significant association (p = 0.011*). Other comorbidities did not exhibit statistically significant relationships, including chronic liver disease (p = 0.054) and hypertension (p = 0.077).

**Table 4:** Categorical Variables Analysis Using Chi-Square Test Odds ratios (OR) provide a clearer effect size interpretation. 95% CI shows the range where the true OR is likely to fall. *p-values <0.05 indicate significant associations.

Variable	Category	Frequency (n; %)	Chi-Square Test (χ²)	Odds Ratio (OR)	95% CI	p-value (<0.05)
Gender	Male	194 (59.51)	1.89	1.12	(0.89–1.45)	0.17
	Female	132 (40.49)				
Comorbidities	Hypertension	120 (36.80)	3.12	1.24	(0.91–1.68)	0.077
	Diabetes	98 (30.06)	-	1.15	(0.82–1.58)	-
	Chronic Kidney Disease	72 (22.08)	6.43	2.01	(1.27–3.15)	0.011*
	Chronic Liver Disease	54 (16.56)	3.71	1.49	(0.98–2.28)	0.054
Mortality (30-day)	Yes	46 (14.11)	16.26	3.75	(2.41–5.83)	<0.001*
	No	280 (85.89)				

The findings of the multivariate regression analysis identifying factors influencing clinical outcomes in sepsis patients are presented in Table 5. Vitamin C supplementation was significantly associated with improved outcomes (β = -2.34, 95% CI: -4.12 to -0.56, p = 0.012, OR = 0.76, 95% CI: 0.59-0.92), indicating a protective effect. Age (β = 0.05, 95% CI: 0.01 to 0.09, p = 0.020, OR = 1.05, 95% CI: 1.01-1.09), SOFA score (β = 0.34, 95% CI: 0.15 to 0.53, p < 0.001, OR = 1.40, 95% CI: 1.21-1.58), and APACHE II score (β = 0.47, 95% CI: 0.27 to 0.68, p < 0.001, OR = 1.60, 95% CI: 1.31-1.92) were also significant predictors of worse outcomes.

**Table 5:** Multivariate Regression Analysis for Clinical Outcomes P-values <0.05 were considered significant. The model was adjusted for age, SOFA score, APACHE II score, and comorbidities (hypertension, diabetes, and chronic kidney disease). β coefficient: Represents the estimated change in the dependent variable per unit increase in the independent variable. 95% CI: Indicates the range within which the true effect size is likely to fall. Standardized β coefficients: Allow comparison of the relative impact of different variables. *p-values: Determine statistical significance (p < 0.05 significant). Odds Ratios (OR) or Hazard Ratios (HR): Estimate effect size and event likelihood.

Variable	Coefficient (β)	95% Confidence Interval	Standardized β	p-value (<0.05)	Odds Ratio (OR) / Hazard Ratio (HR)
Vitamin C Supplementation	-2.34	-4.12 to -0.56	-0.28	0.012*	0.76 (0.59–0.92)
Age (years)	0.05	0.01 to 0.09	0.12	0.020*	1.05 (1.01–1.09)
SOFA Score	0.34	0.15 to 0.53	0.42	<0.001*	1.40 (1.21–1.58)
APACHE II Score	0.47	0.27 to 0.68	0.38	<0.001*	1.60 (1.31–1.92)
Chronic Kidney Disease	1.75	0.87 to 2.63	0.24	<0.001*	5.75 (3.42–9.62)
Sepsis Source (Pulmonary)	0.89	0.43 to 1.35	0.16	0.045*	2.44 (1.62–3.69)

Among comorbidities, chronic kidney disease was strongly correlated with adverse outcomes (β = 1.75, 95% CI: 0.87 to 2.63, p < 0.001, OR = 5.75, 95% CI: 3.42-9.62), highlighting its impact on sepsis progression. In contrast, diabetes (β = 0.98, 95% CI: 0.41 to 1.55, p = 0.124, OR = 2.44, 95% CI: 1.62-3.69) and hypertension (β = 1.20, 95% CI: 0.55 to 1.86, p = 0.164) were not statistically significant predictors. These findings emphasize the therapeutic potential of vitamin C in sepsis, demonstrating its significant role in improving clinical outcomes. These findings (Table 5) confirm that disease severity and chronic conditions are key factors influencing patient prognosis.

## Discussion

The study's findings show that vitamin C supplementation had a major impact on sepsis patients' clinical outcomes. In particular, our results showed that over a one-year period, organ dysfunction ratings decreased while the mean ICU stay and death rates improved. After improving to 7.86 ± 3.52 at three months, 6.93 ± 3.15 at six months, 5.68 ± 2.87 at nine months, and 4.83 ± 2.54 at 12 months, the mean organ dysfunction score at baseline was 9.82 ± 4.03. These results are in line with other studies that show how vitamin C helps lower oxidative stress and inflammatory markers, which in turn improves sepsis clinical outcomes [16,17].

Our research found that C-reactive protein (CRP) levels significantly decreased from a baseline mean of 175.31 ± 62.43 mg/L to 154.67 ± 49.87 mg/L at three months, 134.22 ± 43.11 mg/L at six months, and 114.57 ± 38.67 mg/L at 12 months, suggesting a considerable reduction in inflammatory markers. Pro-inflammatory cytokines like TNF-α and IL-6 also declined; at baseline, IL-6 was 345.16 ± 128.52 pg/mL; at three months, it was 305.14 ± 112.74 pg/mL; at six months, it was 275.13 ± 96.26 pg/mL; and at 12 months, it was 245.12 ± 83.78 pg/mL. These decreases in inflammatory markers are consistent with research showing that vitamin C administration lowers systemic inflammation in individuals with sepsis [18].

From 8.94 ± 3.21 nmol/mL at baseline to 7.89 ± 2.87 nmol/mL at three months, 6.84 ± 2.53 nmol/mL at six months, and 5.79 ± 2.18 nmol/mL at 12 months, we found a steady drop in oxidative stress indicators, especially MDA. The antioxidant qualities of vitamin C have been shown in earlier studies to lessen oxidative stress, which is a major cause of tissue damage and organ dysfunction in sepsis [19,20].

The 30-day mortality rate in our research was 14.11% at baseline, but it gradually dropped to 12.50%, 10.30%, 8.70%, and 6.90% after 3, 6, 9, and 12 months, respectively. These findings are consistent with earlier research showing that vitamin C administration reduces oxidative stress and inflammation, which in turn lowers death rates in sepsis patients [21,22].

These results align with previous studies demonstrating that vitamin C reduces oxidative stress and inflammatory markers, thereby improving clinical outcomes in sepsis. However, our findings contrast with the results of the LOVIT trial, a multicenter RCT that reported increased mortality and organ dysfunction at 28 days following a 4-day course of intravenous vitamin C [23]. Notably, a secondary analysis of the same trial found that the elevated mortality may have resulted from the abrupt discontinuation of vitamin C rather than its administration itself [24]. Our study’s sustained supplementation and observed long-term improvements suggest that treatment duration and tapering strategies may play a critical role in patient outcomes. These discrepancies highlight the need for future randomized controlled trials to clarify optimal dosing duration and withdrawal protocols for vitamin C therapy in sepsis patients.

Overall, the findings of our investigation align with other research indicating that vitamin C administration improves oxidative stress, inflammatory markers, and sepsis clinical outcomes. The long-term potential advantages of vitamin C treatment in treating sepsis-related sequelae are highlighted by the consistent decline in organ dysfunction, inflammatory markers, and death rates.

Study strengths and limitations

This study has several strengths. It is one of the few to evaluate the long-term effects of vitamin C supplementation in sepsis, offering valuable insights into its role in modulating inflammation, oxidative stress, and clinical outcomes. The prospective observational design and relatively large sample size (n = 326) enhance the generalizability and reliability of the findings. Rigorous data collection included key inflammatory and oxidative stress biomarkers, allowing for a more comprehensive understanding of vitamin C's effects on sepsis-related pathophysiology. Additionally, multivariate regression analysis was performed to identify independent determinants of clinical outcomes, further strengthening the validity of the results.

To minimize bias, a consecutive sampling approach was used, ensuring that all eligible patients were enrolled without pre-selection. Standardized treatment protocols were followed, and clinical outcomes were assessed through structured follow-ups at 3, 6, 9, and 12 months. Patient data were anonymized, and statistical adjustments were made to account for potential confounders.

Despite these strengths, the study has some limitations. As an observational study, it cannot establish a direct causal relationship between vitamin C supplementation and improved outcomes. Although statistical adjustments were made, residual confounding due to variations in baseline patient characteristics and comorbidities remains a possibility. The lack of a randomized control group limits the ability to completely isolate the effects of vitamin C from other concurrent therapies. Additionally, while the vitamin C dosing regimen was standardized, individual patient responses may vary. Future randomized controlled trials are needed to validate these findings and refine optimal dosing strategies.

## Conclusions

The baseline demographic and clinical characteristics of sepsis patients showed that most were middle-aged with a predominance of males and commonly presented with comorbidities such as hypertension, diabetes, chronic kidney disease, and chronic liver disease. High APACHE II and SOFA scores at admission indicated severe illness, while significantly elevated inflammatory markers (CRP, TNF-α, IL-6) and oxidative stress marker (MDA) reflected systemic inflammation and oxidative damage. ICU stays were prolonged with notable organ dysfunction and a substantial 30-day mortality rate. Over a 12-month follow-up, patients - especially those receiving vitamin C supplementation - demonstrated a consistent, statistically significant improvement in ICU stay duration, organ dysfunction scores, and mortality, with stepwise recovery across clinical indicators. Chi-square analysis revealed a significant association between 30-day mortality and chronic kidney disease, but not with gender or other comorbidities. These findings underscore the heavy clinical and biochemical burden of sepsis while suggesting that adjunctive vitamin C supplementation may offer therapeutic benefit by reducing inflammation, oxidative stress, and early mortality. Future randomized controlled trials (RCTs) are warranted to confirm these observational findings and to establish standardized protocols for the use of vitamin C and other antioxidant therapies in sepsis management.
